# Glutamine metabolism regulates endothelial to hematopoietic transition and hematopoietic lineage specification

**DOI:** 10.1038/s41598-021-97194-7

**Published:** 2021-09-02

**Authors:** Leal Oburoglu, Els Mansell, Niels-Bjarne Woods

**Affiliations:** Molecular Medicine and Gene Therapy, Lund Stem Cell Center, Lund University, BMC A12, 221 84 Lund, Sweden

**Keywords:** Metabolic pathways, Haematopoietic stem cells, Pluripotent stem cells, Stem-cell differentiation, Haematopoiesis

## Abstract

During hematopoietic development, definitive hematopoietic cells are derived from hemogenic endothelial (HE) cells through a process known as endothelial to hematopoietic transition (EHT). During EHT, transitioning cells proliferate and undergo progressive changes in gene expression culminating in the new cell identity with corresponding changes in function, phenotype and morphology. However, the metabolic pathways fueling this transition remain unclear. We show here that glutamine is a crucial regulator of EHT and a rate limiting metabolite in the hematopoietic differentiation of HE cells. Intriguingly, different hematopoietic lineages require distinct derivatives of glutamine. While both derivatives, α-ketoglutarate and nucleotides, are required for early erythroid differentiation of HE during glutamine deprivation, lymphoid differentiation relies on α-ketoglutarate alone. Furthermore, treatment of HE cells with α-ketoglutarate in glutamine-free conditions pushes their differentiation towards lymphoid lineages both in vitro and in vivo, following transplantation into NSG mice. Thus, we report an essential role for glutamine metabolism during EHT, regulating both the emergence and the specification of hematopoietic cells through its various derivatives.

## Introduction

During development, hematopoiesis occurs in three consecutive waves. The first wave, termed primitive hematopoiesis, mainly gives rise to primitive erythroid cells and macrophages^1^. The second wave is characterized as definitive and allows the formation of mature erythroid, myeloid and lymphoid cells^2,3^. Lastly, the third hematopoietic wave gives rise to hematopoietic stem cells (HSCs)^4,5^ which will then proliferate in the fetal liver and migrate to the bone marrow (BM) to act as lifelong providers of hematopoietic cells. All hematopoietic cells derived in the three hematopoietic waves arise from endothelial cells^6–8^ and definitive hematopoietic development starts with a specialized set of endothelial cells with hemogenic potential that differentiate towards hematopoietic cells through a process known as endothelial to hematopoietic transition (EHT)^9,10^. The in vitro differentiation from induced pluripotent stem cells (iPSCs) appears to recapitulate aspects of primitive and definitive hematopoietic waves, with the generation of both primitive erythroid cells expressing embryonic and fetal globins, as well as lymphoid cells among other hematopoietic cells^11–14^, making it an ideal system to study specific regulators involved in human hematopoietic cell development. The EHT process has been shown to rely on various signaling pathways and the expression of specific transcription factors^15–17^. Nevertheless, the influence of nutrients and metabolic pathways during hematopoietic cell development from endothelial cells have not been sufficiently studied to this day.

Glutamine, the most abundant amino acid in blood plasma, is an essential precursor for the tricarboxylic acid (TCA) cycle (also known as Krebs cycle, or citric acid cycle), which is centrally important for cellular respiration and energy production^18^. By giving rise to α-ketoglutarate (α-KG), glutamine feeds the TCA cycle by anaplerosis and consequently fuels oxidative phosphorylation (OXPHOS) for ATP production^19^. Moreover, glutamine also participates in several other metabolic pathways including nucleotide biosynthesis and non-essential amino acid (NEAA) synthesis^20^. Through its contribution to all these pathways, glutamine acts as a crucial bioenergetic fuel and building block, especially in rapidly proliferating cells^19^. Incidentally, it has been shown that committed hematopoietic progenitors arising from the hemogenic endothelium (HE) increase their proliferative rates in the newly forming hematopoietic clusters^21,22^, which suggests that glutamine may be involved in this process. In this study, we describe an important role for glutamine during EHT, both for the proliferation of transitioning cells and their differentiation into distinct hematopoietic lineages.

## Results

### Glutamine is a fuel for the TCA cycle and OXPHOS for hematopoietic cell differentiation from HE

**Figure 1:**
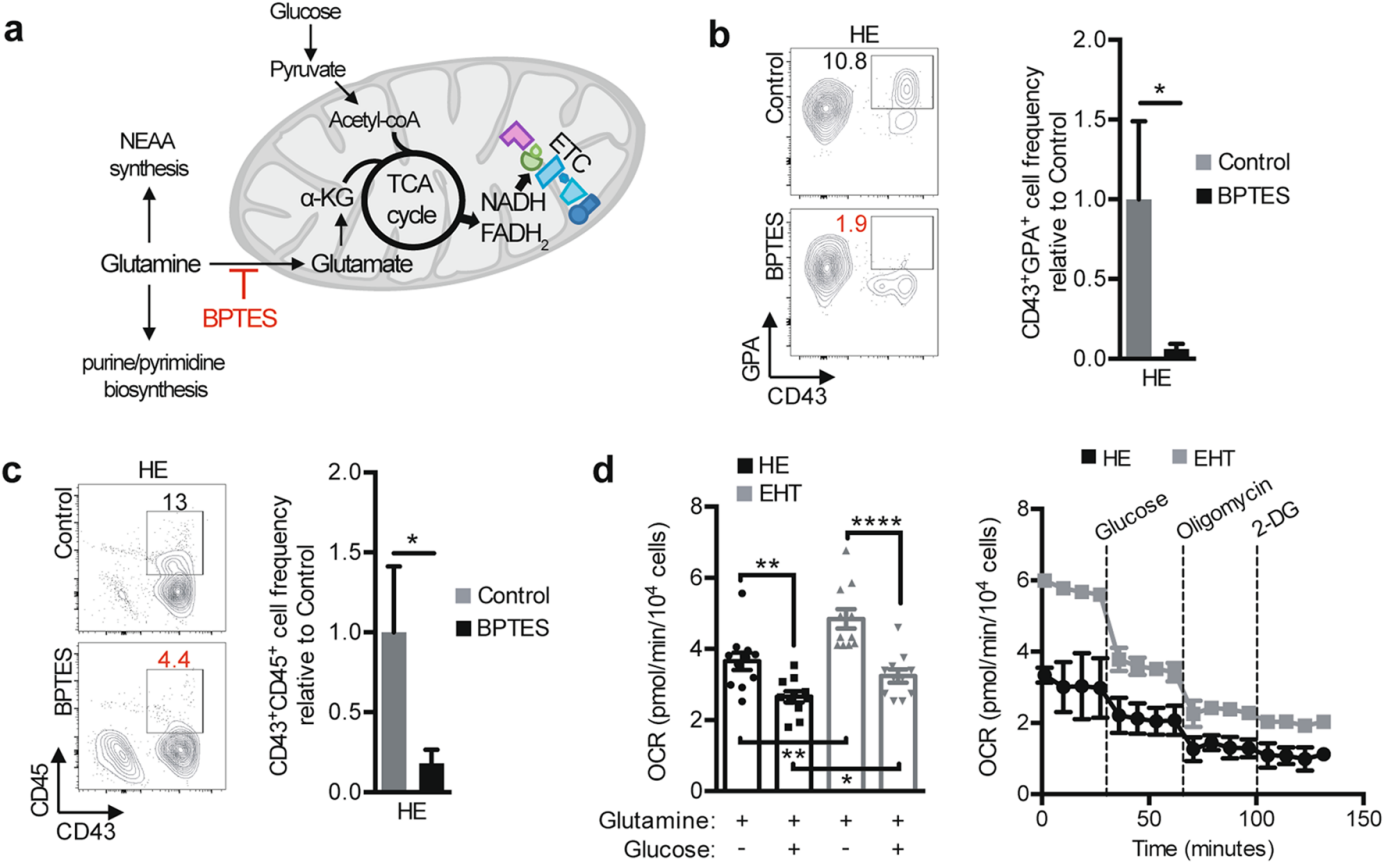
The entry of glutamine into the TCA cycle is required for hematopoietic differentiation of HE cells. (**a**) Schematic showing the contribution of glutamine to various biosynthetic pathways. In mitochondria, glutamine is deamidated to glutamate (Glu) which is then converted to α-ketoglutarate (α-KG), an intermediate of the TCA cycle. The conversion of glutamine to glutamate is mediated by the glutaminase (GLS) enzyme, which is specifically inhibited by BPTES. In the cytosol, glutamine is used to for purine and pyrimidine biosynthesis, as well as non-essential amino acid (NEAA) synthesis. ETC, electron transport chain. (**b** and **c**) Day 8 FACS-sorted HE cells were subcultured with or without BPTES (25 µM). Subculture day 3 representative plots and bar graphs (**b**) show CD43^+^GPA^+^ cell frequency ± s.e.m. (n = 6, paired *t*-tests). Subculture day 6 representative plots and bar graphs (**c**) show CD43^+^CD45^+^ cell frequency ± s.e.m. (n = 4, paired *t*-tests). (**d**) OCR was measured in HE and EHT cells (n = 11) in the absence of glucose as well as after the injection of the indicated compounds and a representative flux graph is shown. Corresponding bar graphs show mean levels ± s.e.m. of OCR in the absence of glucose and after glucose injection (n = 11, from 3 independent experiments, unpaired *t*-tests). *p < 0.05, **p < 0.01, ****p < 0.0001.

To model endothelial to hematopoietic transition in the human setting, hiPSCs were differentiated as previously described^23^ and both HE as well as cells undergoing EHT (which we have termed EHT cells) were isolated based on previously described FACS phenotypes with HE and EHT cells defined as CD34^+^CD43^−^CXCR4^−^CD73^−^CD90^+^VECad^+^ and CD34^+^CD43^int^CXCR4^-^CD73^-^CD90^+^VECad^+^ cells, respectively^23,24^. To determine whether glutamine is important for fueling the TCA cycle during EHT, we blocked the glutaminase (GLS) enzyme, which catalyzes the deamidation of glutamine to glutamate, by treating HE cells with an allosteric glutaminase inhibitor, bis-2-(5-phenylacetamido-1,3,4-thiadiazol-2-yl)ethyl sulfide (BPTES) (Fig. 1a). Sorted HE cells were subcultured with BPTES for 3–6 days in hematopoietic differentiation medium and evaluated for hematopoietic lineage output. Previously, we and others have demonstrated an early erythroid population of cells expressing both the human early pan-hematopoietic cell marker, CD43, and the erythroid marker glycophorin A (GPA), arising from HE or similar cells at day 3 of the protocol, which predominantly expresses fetal and embryonic globins^12,14,24^. While hematopoietic cells harvested at day 6 expressing the markers CD43^+^CD45^+^ corresponded to a population with lymphoid and myeloid cell potential^12^. In the presence of BPTES, we observed a significant decline in the generation of both the HE-derived CD43^+^GPA^+^ erythroid population at day 3 (Fig. 1b), as well as the CD43^+^CD45^+^ population at day 6 (Fig. 1c). This result suggests that the entry of glutamine into the TCA cycle is required for both early (erythroid, day 3) and late (CD45^+^, day 6) hematopoietic cell specification during EHT.

To test whether HE and EHT cells rely on glutamine for energy production via OXPHOS, we measured the oxygen consumption rate (OCR) of these cells by extracellular flux analysis. Interestingly, in medium with glutamine without glucose, both HE and EHT cells consumed oxygen at higher levels compared to medium with both glutamine and glucose (Fig. 1d, bar graphs). The high OCR levels in both HE and EHT cells before glucose injection during the extracellular flux assay shows that the cells efficiently consume oxygen with glucose-free glutamine supplemented medium compared to medium with both glutamine and glucose, as this resulted in a switch to glycolysis (Fig. 1d, flux graph). Thus, even in glucose-free medium, HE and EHT cells had high basal respiration levels, suggesting that these cells utilize glutamine for the TCA cycle and mitochondrial respiration. Moreover, we detected a higher basal respiration rate in EHT cells as compared to HE cells, regardless of the presence or absence of glucose in the medium (Fig. 1d), showing that OXPHOS rates increase during EHT and that glutamine consumption may be sufficient to fuel this process.

### Glutamine is the rate limiting step in hematopoietic cell differentiation from HE

**Figure 2:**
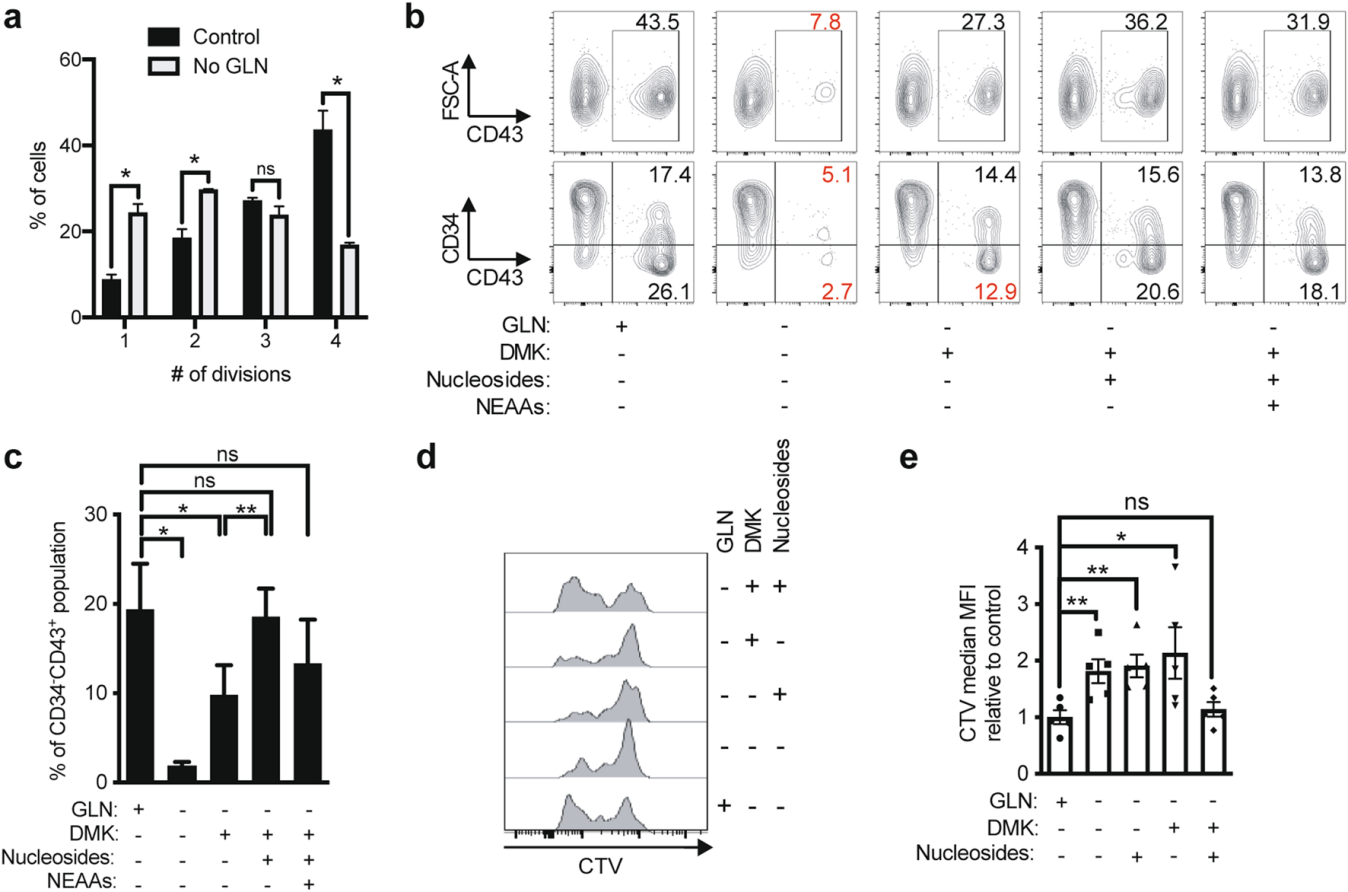
Glutamine is essential for the proliferation of differentiating cells during EHT. (**a**) Day 8 FACS-sorted EHT cells were stained with CellTrace Violet (CTV) proliferation dye and subcultured with or without glutamine (2 mM). For each division stage, the percentage of EHT-derived CD43^+^ cells ± s.e.m. at day 3 are shown (n = 2, unpaired *t*-tests). See representative plots in Fig. S2b. (**b**–**e**) FACS-sorted HE cells were subcultured in glutamine-free medium with the indicated compounds: DMK (1.75 mM), Nucleosides or NEAAs. (**b**, **c**) Subculture day 3 representative plots showing the expression of FSC-A*/*CD43 and CD34*/*CD43 are shown (n = 3, see (**c**) for bar graphs). (**d**, **e**) Subculture day 3 CellTrace Violet (CTV) fluorescence was assessed in total cells obtained from HE cells by flow cytometry (n = 4, see (**e**) for bar graphs). ns, not significant, *p < 0.05, **p < 0.01.

To get a better grasp of the role of glutamine during EHT, we cultured HE and EHT cells in glutamine-free medium. Intriguingly, while the absence of glutamine was not toxic for HE cells, it led to significantly fewer cells in EHT cell cultures, with > 40% decrease in live cells at day 3 of subculture (Fig. S1a). To understand whether this was due to a difference in proliferation, we assessed the number of divisions EHT-derived CD43^+^ cells have gone through after 3 days of subculture. We stained EHT cells with the Cell Trace Violet (CTV) dye which allows to follow proliferation, as it dilutes with each division. We show that while most EHT-derived CD43^+^ cells have divided 4 times in the control condition, these cells only divided 1–2 times in the absence of glutamine (Fig. 2a and Fig. S1b). Thus, glutamine is important for the increasing energy demands of the proliferating EHT-derived CD43^+^ cells, and may also be required for newly committed hematopoietic cells which may not be sustained in glutamine-free medium. Indeed, the overriding effect of glutamine deprivation was a significant reduction in the acquisition of the CD43^+^ hematopoietic cell marker (> 80% decrease) from HE cells at day 3 of subculture (Fig. 2b), likely a culmination of effects on the newly emerged hematopoietic cells themselves.

To understand which metabolic pathway involving glutamine is important for the hematopoietic differentiation of HE and to rescue this phenotype, we supplemented the glutamine-free culture medium with the metabolites and substrates from three molecular pathways for energy and biochemical production from glutamine^18^ (Fig. 1a); nucleosides, non-essential amino acids (NEAAs), or a cell-permeable form of α-ketoglutarate (dimethyl-ketoglutarate, DMK). Nucleosides, NEAAs or a combination of both could not rescue the effect seen in glutamine deprivation (Fig. S2a). However, DMK addition rescued total CD43^+^ cell output from HE cells up to 60% (Fig. 2b, c). Moreover, a combination of DMK/nucleosides, or DMK/nucleosides/NEAAs further increased the percentage of CD34^-^CD43^+^ cells deriving from HE cells, reaching the levels in the control condition (Fig. 2c). These results demonstrate that all these metabolic pathways of glutamine use are required for optimal hematopoietic cell differentiation from HE, where fueling the TCA cycle (via DMK) is most critical.

To assess whether pyruvate, another fuel for the TCA cycle, could replace glutamine, we treated HE cells with a pyruvate dehydrogenase kinase (PDK) inhibitor, dichloroacetate (DCA), to increase pyruvate dehydrogenase (PDH) activity during glutamine deprivation. We found that without glutamine, DCA treatment alone could not restore the CD43^+^ cell levels seen in the control and rescue the absence of glutamine (Fig. S2b), emphasizing the requirement for glutamine to fuel the TCA cycle in the energy demanding process of hematopoietic cell differentiation from HE.

Given the above findings that glutamine fueling the TCA cycle and nucleotide production was required for hematopoietic cell (CD43^+^) production in general, we further analyzed subpopulations of cells undergoing EHT and hematopoietic cell expansion to determine which glutamine derivatives were required for which hematopoietic populations. Intriguingly, subpopulations of cells within the CD43^+^ fraction were differentially affected by the different glutamine derivatives. In particular, the percentage of more mature CD43^+^ cells that have lost CD34^+^ expression was significantly decreased both in the glutamine-free and the glutamine-free DMK-treated condition (TCA cycle rescue only) as compared to the control (Fig. 2b, c). However, nucleoside addition alone or together with NEAAs restored the percentage of CD43^+^CD34^-^ cells in the glutamine-free DMK-treated conditions to the levels observed in the control (Fig. 2c). As nucleotides are essential in proliferating cells for DNA synthesis, we hypothesized that the proliferation of differentiating hematopoietic cells depended on this factor, where absence of this factor blocks hematopoietic progenitor differentiation. We observed that DMK or nucleosides alone could not restore the proliferation profile seen in the control condition; indeed, only the addition of both these factors reinstated the proliferation of HE cells during glutamine deprivation (Fig. 2d, e). Taken together, these results indicate that glutamine is essential for fueling TCA cycle for the process of EHT and early hematopoietic progenitor production, and also for contributing to nucleotide production for proliferative support in HE-derived hematopoietic progenitors during their differentiation towards committed hematopoietic cells.

### Glutamine differentially sustains hematopoietic populations

**Figure 3:**
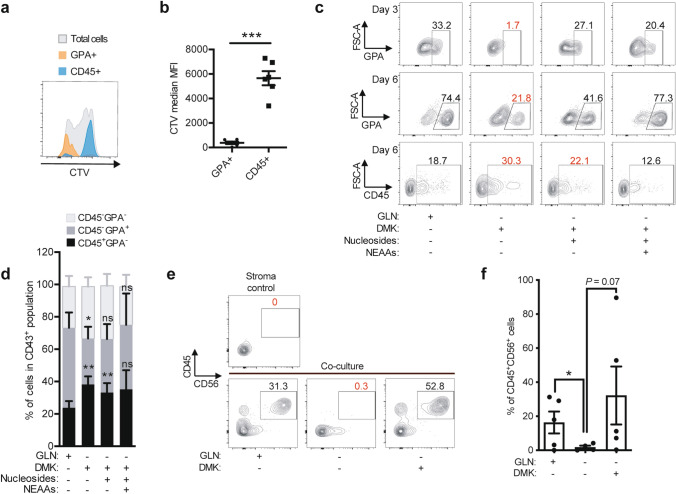
Glutamine differentially contributes to the formation of GPA^+^ and CD45^+^ cells during EHT. (**a**) Representative day 3 CellTrace Violet (CTV) plot is shown for GPA^+^ (in orange) and CD45^+^ (in blue) populations deriving from HE cells. (**b**) CTV median MFIs ± s.e.m. corresponding to (**a**) are shown (n = 6, paired *t*-tests). (**c**, **d**) FACS-sorted HE cells were subcultured in glutamine-free medium with the indicated compounds: DMK (1.75 mM), Nucleosides or NEAAs. (**c**) Subculture day 3 or day 6 representative plots, gated on the CD43^+^ population, are shown for FSC-A*/*GPA and FSC-A/CD45. (**d**) Percentages of cells expressing GPA or CD45 in the CD43^+^ population ± s.e.m. at day 6 of subculture are depicted (Control, n = 7; DMK, n = 6; DMK + nucleosides, n = 5; DMK + nucleosides + NEAAs, n = 3; paired *t*-tests with the controls). (**e**, **f**) FACS-sorted HE cells were subcultured in glutamine-free medium with or without DMK (1.75 mM) for 3 days, after which the treatment was removed and the cells were co-cultured with OP9-DL1 stroma for 35 days to induce NK cell differentiation. (**e**) Plots showing percentages of CD45^+^CD56^+^ (NK) cells obtained at day 35. (**f**) Percentages of CD45^+^CD56^+^  ± s.e.m. cells obtained at day 35 (Control and DMK, n = 5; −GLN, n = 4). ns, not significant, *p < 0.05, **p < 0.01, ***p < 0.001.

Previously, erythroid differentiation of highly proliferative cord blood CD34^+^ progenitors has been shown to require glutamine-fueled nucleotide synthesis^25^. To further understand the role of glutamine derivatives fueling EHT from HE cells, we also investigated the CD43^+^GPA^+^ erythroid-committed and CD45^+^ (lymphoid/myeloid potential) populations in this context. First, we stained HE cells with CTV and assessed the proliferation status of newly-formed GPA^+^ or CD45^+^ cells 3 days later. While GPA^+^ cells clustered to the divided cells (low CTV MFI values), interestingly, CD45^+^ cells deriving from HE cells had few divisions (high CTV MFI values; Fig. 3a, b). The clear separation between these populations based on their proliferation history prompted us to then investigate whether they have different requirements in terms of glutamine-derived factors. At days 3 and 6 of the HE subcultures without glutamine, DMK alone could not rescue the CD43^+^GPA^+^ population to the levels seen in the control (Fig. 3c). However, a combination of DMK/nucleosides or DMK/nucleosides/NEAAs gave rise to a CD43^+^GPA^+^ population comparable to the one seen in the presence of glutamine (Fig. 3d). Consequently, glutamine acts as both a carbon- and nitrogen-donor to produce α-ketoglutarate and nucleotides, which are both required for the production of CD43^+^GPA^+^ from HE cells, in line with their proliferation profile (Fig. 3a).

Interestingly, we observed a significant increase in the percentages of CD43^+^CD45^+^ cells in conditions restored by DMK or DMK/nucleosides (Fig. 3c, d) compared to the control. In line with our finding that HE-derived CD45^+^ cells are slower to initiate proliferation (Fig. 3a), DMK was sufficient for their derivation from HE even in the absence of nucleosides. Thus, a boost in the TCA cycle using DMK favors the formation of CD45^+^ hematopoietic cells.

The CD45 marker is expressed by all mature hematopoietic cells, with the exception of mature erythroid cells^26,27^. As lymphoid cell formation in vitro requires co-culture experiments, in order to understand whether DMK induces the formation of lymphoid hematopoietic cells, we co-cultured day 3 HE cells with OP9-DL1 stroma and induced lymphoid cell differentiation. While glutamine deprivation during the 3-day subculture period prevented HE cells from being able to give rise to lymphoid natural killer (NK) cells later in co-cultures, DMK supplementation allowed for efficient NK cell differentiation (Fig. 3e, f). Therefore, the entry of glutamine into the TCA cycle is sufficient to induce lymphoid cell differentiation from HE cells. Taken together, these results show that glutamine use for the TCA cycle is essential during EHT for differentiation of HE to form CD45^+^ cells including lymphoid lineage (NK) potentiated cells, and that glutamine fueling both TCA cycle and nucleotide production is essential for formation and expansion of the highly proliferative early (day 3) erythroid (GPA^+^) cells.

### Exclusive use of glutamine for the TCA cycle favors lymphoid differentiation of HE in vivo

**Figure 4:**
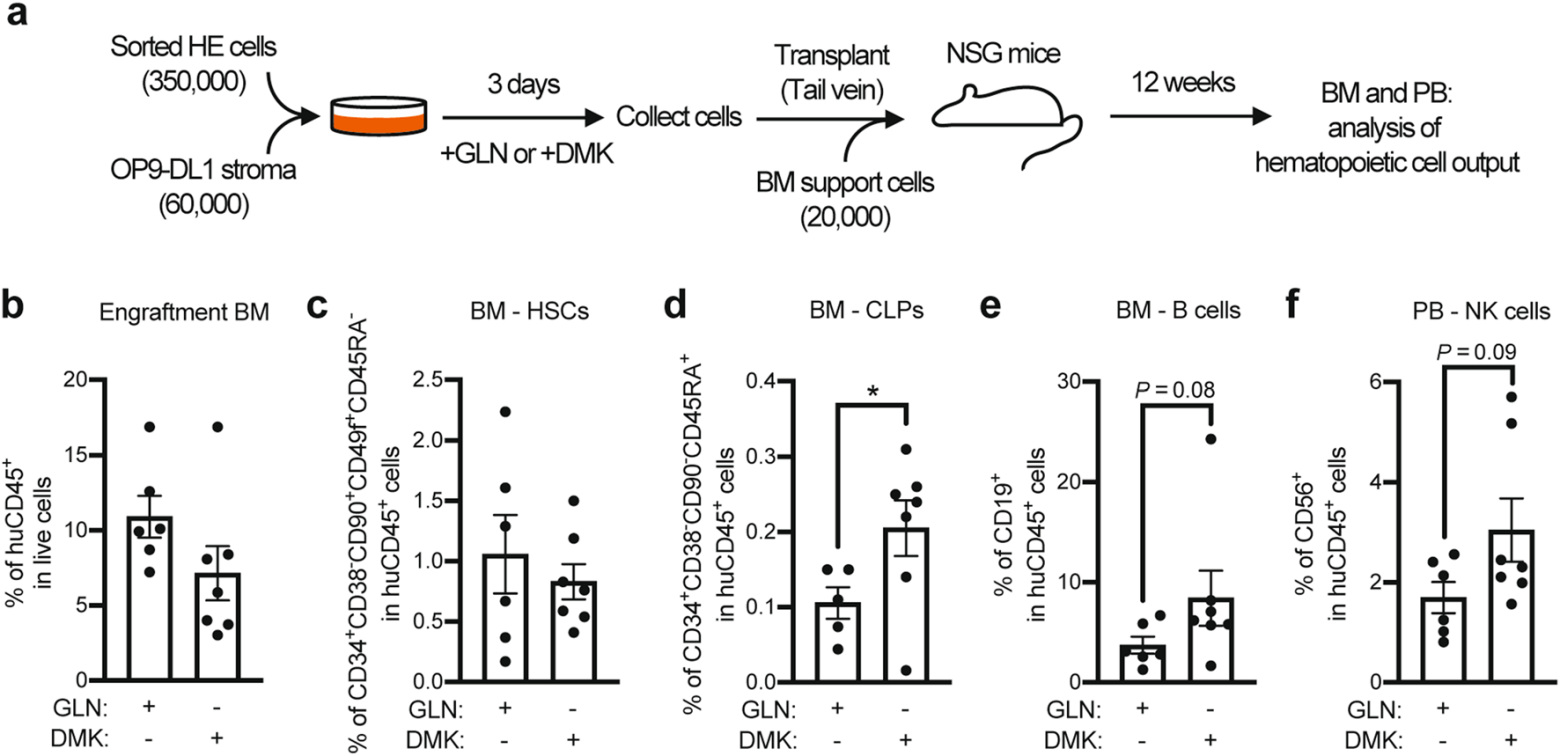
Boosting glutamine use for the TCA cycle via DMK in HE cells favors lymphoid repopulation in NSG mice. (**a**) Experimental design for in vivo assessment of HE cell potential. HE cells were co-cultured with OP9-DL1 stroma with glutamine (+GLN) or in glutamine-free medium with 1.75 mM DMK (+DMK) for 3 days and transplanted into sub-lethally irradiated NSG mice. Bone marrow (BM) and peripheral blood (PB) were harvested on week 12. (**b**) Engraftment levels in BM as percentages of huCD45^+^ cells are shown (+GLN, n = 6; +DMK, n = 7; from 2 independent experiments). The percentages ± s.e.m. of human HSCs (**c**), CLPs (**d**) and B cells (**e**) in huCD45^+^ cells from the BM are shown (+GLN, n = 6; +DMK, n = 7; unpaired *t* tests). (**f**) The percentages ± s.e.m. of human CD56^+^ NK cells in huCD45^+^ cells from the PB are shown (+GLN, n = 6; +DMK, n = 7; from 2 independent experiments; unpaired *t* tests). *p < 0.05.

As we detected an enhanced differentiation of DMK-treated HE cells in glutamine-free medium towards a CD45^+^ fate (Fig. 3c) and an increased lymphoid lineage potentiation (Fig. 3e) compared to standard glutamine conditions, we sought to evaluate this result in an in vivo setting. Using immunocompromised (NSG) mice, which specifically allows to evaluate repopulating hematopoietic cells excluding cells from the mature erythroid lineage, we could obtain a clear picture of the role of DMK in lymphoid potentiation of HE-derived repopulating cells. We co-cultured glutamine-deprived DMK-treated HE cells with OP9-DL1 stroma for 3 days, before transplanting the bulk cells into sub-lethally irradiated NSG mice to evaluate the hematopoietic potential of HE cells in vivo (Fig. 4a). The OP9-DL1 stroma was used to provide Notch signaling to increase the likelihood of generating definitive repopulating cells, as previously demonstrated^28,29^. At 12 weeks, we observed a similar BM engraftment level for both control and glutamine-deprived DMK-treated HE cells (Fig. 4b). This result demonstrates that glutamine-fueled nucleotide production is not a requirement for generating repopulating cells during EHT, but rather the primary function of glutamine towards the production of in vivo repopulating cells is to fuel the TCA cycle. Therefore, and as expected from the above result, when we then evaluated the frequency of phenotypic HSCs within the BM of the transplanted mice, we found that they were not significantly different between the two conditions (Fig. 4c). However, there was a significant increase in the frequency of the common lymphoid progenitors (CLPs) in the BM (Fig. 4d). In line with this result, we detected a tendency towards increased B cells deriving from glutamine-deprived DMK-treated HE cells compared to the control (Fig. 4e). Last but not least, we observed a tendency towards increased NK cell formation from glutamine-deprived DMK-treated HE cells compared to the control in peripheral blood (Fig. 4f), in line with our in vitro results (Fig. 3e, f). Altogether, these in vivo results confirm that the use of glutamine for the TCA cycle via α-ketoglutarate favors the differentiation of HE towards lymphoid repopulating cells during EHT.

## Discussion

Our study demonstrates for the first time that glutamine is crucial for the EHT process and regulates both the emergence of hematopoietic cells and their cell fate specification. We show how glutamine plays distinct roles in the specification and expansion of highly proliferative early erythroid cells versus slower proliferating lymphoid-biased hematopoietic cells (including NSG mouse-repopulating cells) from human HE. In developing embryos, primitive erythroid cells were shown to perform high rates of glycolysis to fuel their rapid proliferation^30^. Moreover, a crucial role for glutamine in supplying nucleotides for erythroid differentiation has been previously described in the context of HSCs obtained from cord blood^25^. Similarly, in our setting, the GPA^+^ cells derived from HE proliferate faster than CD45^+^ cells and thus were shown to rely on glutamine for providing nucleotides for this process. On the other hand, we show that anaplerotic entry of glutamine into the TCA cycle is the limiting step for CD45^+^ cell formation from HE. Likewise, dysregulation of the TCA cycle by downregulation of a crucial component, fumarate hydratase, has been previously shown to impair fetal hematopoiesis and specifically multilineage differentiation^31^.

Intriguingly, we show that treatment of HE cells with DMK in glutamine-free medium leads to a significant increase in the formation of CD45^+^ cells and specifically lymphoid cells, both in vitro and in vivo. While in vivo*,* the total engraftment levels were not statistically different between the DMK and glutamine conditions, the hematopoietic lineage output of repopulating cells was somewhat altered. Therefore, we can infer that metabolism can influence cell fate decisions already at the HE and EHT stages, prior to full hematopoietic commitment. This is in line with our and others’ previous findings reporting that lineage propensities differ among EHT and pre-HSC stage populations^24,32^. Furthermore, while we could not conclusively confirm a role of glutamine in definitive HSC repopulation ability, we did confirm the formation of lymphoid (NK and B cells) lineage differentiation of transitioning cells via glutamine modulation, which may either be cells of definitive or earlier waves of hematopoiesis. Regardless, development of strategies for generating lymphoid lineage cells with high proliferation capacities is critical to the advancement of iPSC-based lymphoid cell therapies, such as CAR-T or CAR-NK cells.

The TCA cycle component α-ketoglutarate is not only a fuel for the TCA cycle but it also acts as a co-factor for ten-eleven translocation (TET) DNA demethylases^33^. Specifically, TET2 has been extensively shown to play a crucial role in both normal and malignant hematopoiesis^34^. Interestingly, in zebrafish, TET2/3 enzymes were reported to be implicated in the regulation of Notch signaling, which is essential for lymphoid differentiation^35^. These findings could explain the increase we see in lymphoid cell formation from DMK-treated HE cells. Therefore, it will be of interest to further study the role of α-ketoglutarate and TET enzymes in the formation of lymphoid cells from HE.

We show here that glutamine-free conditions abrogate blood emergence from iPSC-derived HE cells. Previously, glutamine deprivation was also shown to impair iPSC-derived mesoderm formation, which is the precursor germ layer for blood^36^. In contrast, cardiomyocytes, another mesoderm-derived cell type, have been shown to heavily rely on glucose rather than glutamine to fuel OXPHOS during their differentiation from iPSCs^37^. This suggests that glutamine is essential for the mesoderm at earlier stages of iPSC culture for hematopoietic differentiation, prior to the formation of endothelial cells, and that metabolic requirements during iPSC differentiation can be significantly different for the formation of various cell types and at each developmental stage.

In conclusion, our study reveals an essential role for glutamine in regulating EHT, hematopoietic cell emergence and differentiation during development. We propose, based on both our in vitro and in vivo results, that the use of glutamine for the TCA cycle via α-ketoglutarate supports the differentiation of HE towards non-erythroid hematopoietic lineages including lymphoid lineages during EHT. We anticipate that adequate manipulation of glutamine metabolism in the context of hematopoietic differentiation of hiPSCs could ultimately facilitate the in vitro generation of specific transplantable hematopoietic cells with therapeutic potential.

## Methods

### hiPSC culture and hematopoietic differentiation

The hiPSC line RB9-CB1 (developed in our laboratory and authentication described in Woods et al*.*^38^) was maintained in culture on feeders (mouse embryonic fibroblasts, Merck-Millipore) and converted to embryoid bodies (EBs) as described previously^24^. The EBs were treated with defined media^23^ to induce hematopoietic differentiation. The stepwise treatment consisted in 2 days with “SFD medium” supplemented with 1 ng/ml Activin A, followed by 4 days with “Day3-SP34 medium” and 2 days with “Day6-SP34 medium”^23^. On days 2 and 3, CHIR99021 (3 µM) was added to the medium. At day 8, EBs were processed to obtain a single cell suspension using TryPLE Express (Thermo Fisher Scientific) from which a CD34^+^ enriched population was isolated using the human CD34 MicroBead kit (Miltenyi). Enriched cells were stained with the following antibodies: CD34-FITC, CD73-PE, VECad-PerCPCy5.5, CD38-PC7, CD184-APC, CD45-AF700, CD43-APCH7, GPA-eF450, CD90-BV605 and 7AAD. The HE (CD34^+^CD43^−^CXCR4^−^CD73^−^CD90^+^VECad^+^) and EHT (CD34^+^CD43^int^CXCR4^−^CD73^−^CD90^+^VECad^+^) cells were sorted using previously defined cell surface markers^24,39,40^ with a FACS Aria III sorter (BD Bioscience).

### HE subculture

FACS-sorted HE (40,000) cells were plated onto Matrigel (16 μg/cm^2^, Corning)-coated 96-well flat bottom plates in HE medium^23^ with 1% penicillin–streptomycin solution (Thermo Fisher Scientific) and kept overnight in a humidified incubator at 37 °C, 5% CO2, 4% O_2_. The following day (termed day 0), media was removed and wells were washed twice with PBS. Fresh HE medium was added, together with BPTES (25 µM) or in glutamine-free medium with DMK (1.75 mM, SIGMA), Nucleosides (Cytidine: 7.3 mg/L; Guanosine: 8.5 mg/L; Uridine: 7.3 mg/L; Adenosine: 8 mg/L; Thymidine: 2.4 mg/L; EmbryoMax, Millipore) or NEAAs (Glycine: 7.5 mg/L, L-Alanine: 8.9 mg/L, L-Asparagine: 13.2 mg/L, L-Aspartic acid: 13.3 mg/L, L-Glutamic Acid: 14.7 mg/L, L-Proline: 11.5 mg/L, L-Serine: 10.5 mg/L; MEM Non-Essential Amino Acids Solution, Thermo Fisher Scientific), where indicated. Every 2 days, media was changed and the indicated compounds were added and cells were kept in a humidified incubator at 37 °C, 5% CO2, 20% O_2_ for 3–6 days.

### NK cell differentiation on OP9-DL1 stroma

Following a 3-day subculture with glutamine or in glutamine-free medium with DMK (1.75 mM, SIGMA) and/or Nucleosides (Cytidine: 7.3 mg/L; Guanosine: 8.5 mg/L; Uridine: 7.3 mg/L; Adenosine: 8 mg/L; Thymidine: 2.4 mg/L; EmbryoMax, Millipore), HE cells were collected with StemPro Accutase Cell Dissociation Reagent and seeded onto OP9-DL1 stroma (80% confluent). The OP9-DL1 murine cell line was kindly provided by Dr. Ewa Sitnicka-Quinn (Lund University, Sweden) and its authentication was described in Renoux et al.^41^. The protocol used to induce NK cell differentiation was described previously by Renoux et al.^41^. Briefly, co-cultures were kept in OP9 medium, consisting of OptiMEM medium with Glutamax (Invitrogen) with 10% FCS, 1% penicillin–streptomycin solution (Thermo Fisher Scientific) and 1% 2-mercaptoethanol (Invitrogen) with SCF (10 ng/ml), FLT3-L (10 ng/ml), IL-2 (5 ng/ml), IL-7 (5 ng/ml, first 15 days only) and IL-15 (10 ng/ml). Cells were passaged onto new OP9-DL1 stroma every week. At day 35, cells were stained for human CD45 and CD56 markers and analyzed on a BD LSRFortessa.

### Extracellular flux analyses

Day 8 FACS-sorted HE or EHT cells (≥ 40,000) were plated onto Matrigel (16 μg/cm^2^, Corning)-coated Seahorse XF96 Cell Culture Microplate wells in 3–4 replicates. Oxygen consumption rate (OCR) was measured 2 days after plating, on a Seahorse XF96 analyzer, in XF medium with 2 mM glutamine after 1-h glucose starvation as well as after the injection by extracellular flux assay of 25 mM glucose, 4 µM oligomycin and 50 mM 2-DG. The rates of OCR were measured 4 times before and after each injection. Data was normalized to cell number.

### Flow cytometry analyses

Cells were collected via StemPro Accutase Cell Dissociation Reagent following 2-min incubation at 37 °C and stained with CD34-FITC, CD45-AF700, CD43-APCH7, GPA-eF450 and the viability marker 7AAD and assessed on a BD LSRII. For measuring proliferation, cells were processed with the CellTrace Violet (CTV) kit according to manufacturer’s instructions (Thermo Fisher Scientific) and fluorescence was measured on a BD LSRFortessa. In all experiments, initial gatings on SSC-A/FSC-A, FSC-H/FSC-A, SSC-H/SSC-A and 7-AAD were done to exclude doublets and dead cells and all plots were generated on FlowJo™ Software (for Mac) Version 10. Ashland, OR: Becton, Dickinson and Company; 2019 (https://www.flowjo.com/solutions/flowjo/downloads).

### NSG mice transplantations

A total of 350,000 FACS-sorted human HE cells and 60,000 OP9-DL1 stroma cells were co-cultured during 3 days with 2 mM glutamine or in glutamine-free medium with 1.75 mM DMK, in HE medium^23^ on Matrigel (16 μg/cm^2^, Corning)-coated 12-well plates. A total of 100,000 to 150,000 cells from either condition together with 20,000 whole BM support cells from C57Bl/6.SJL mice (CD45.1+/CD45.2+ , in house breeding), were transplanted into sub-lethally-irradiated (300 cGy) 8-week-old female NOD/Cg-Prkdc^scid^ Il2rg^tm1Wjl^/SzJ mice (NSG, The Jackson Laboratory) through intravenous tail vein injection. Transplanted cells were in single cell suspension, in 250µL PBS with 2% FBS. During 3 weeks after transplantation, the drinking water of transplanted NSG mice was supplemented with ciprofloxacin (125 mg/L, HEXAL) to prevent infection. Mice were housed in a controlled environment with 12-h light–dark cycles with chow and water provided ad libitum. All experiments indicated and animal care were performed in accordance with the Lund University Animal Ethical Committee’s recommendations and comply with ARRIVE guidelines. The experimental protocol was approved by the Lund University Animal Ethical Committee.

### Bone marrow and peripheral blood analysis after NSG mice transplantations

At week 12 after transplantation, peripheral blood (PB) was collected from the tail vein into EDTA-coated microvette tubes and mature erythrocytes were lysed with ammonium chloride solution (STEMCELL technologies). Bone marrow was harvested through crushing with a pestle and mortar following euthanasia by spinal dislocation and the dissection of both right and left femurs, tibias and iliac bones. The BM cells were collected in 20 mL ice-cold PBS with 2% FBS, filtered, washed and lysed for red blood cells (ammonium chloride solution, STEMCELL technologies). The PB and BM cells were then stained for cell surface antibodies for hematopoietic markers and assessed on the FACS AriaIII (BD). In all experiments, initial gatings were done on SSC-A/FSC-A, FSC-H/FSC-A, SSC-H/SSC-A and DAPI to exclude doublets and dead cells, and on huCD45/muCD45.1 for murine cell exclusion on FlowJo Software.

## Supplementary Information

Supplementary Figures.
