# Supplementary material for: Glutamine metabolism regulates endothelial to hematopoietic transition and hematopoietic lineage specification
Source: Sci Rep. 2021 Sep 2;11:17589. doi: 10.1038/s41598-021-97194-7 (PMC8413451; doi:10.1038/s41598-021-97194-7)
Supplement: Supplementary file 1 — Supplementary Figures. [file 41598_2021_97194_MOESM1_ESM.pdf]

Supplementary information

Glutamine metabolism regulates endothelial to hematopoietic transition and hematopoietic lineage specification

Authors

Leal Oburoglu[1*], Els Mansell[1] and Niels-Bjarne Woods[1*]

Affiliations

[1]Molecular Medicine and Gene Therapy, Lund Stem Cell Center, Lund University, BMC A12, 221 84 Lund, Sweden.

[*]Corresponding authors: leal.oburoglu@med.lu.se, niels-bjarne.woods@med.lu.se

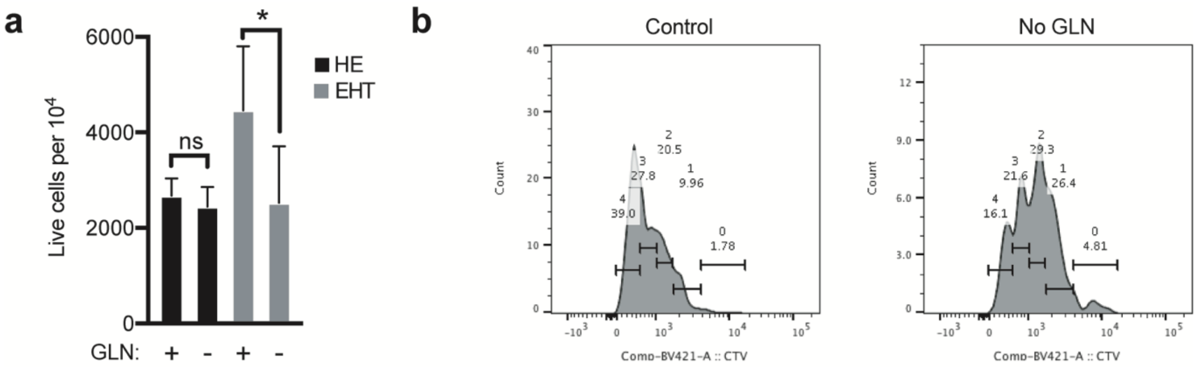

Figure S1. Glutamine is a fuel for proliferation during EHT

(**a**) Day 8 FACS-sorted HE and EHT cells were subcultured with or without glutamine (2 mM). Subculture day 3 bar graphs show live cells (7AAD- singlets) per 10^4 cells ± s.e.m. (HE, n=8; EHT, n=4, paired *t*-tests). (**b**) Day 8 FACS-sorted EHT cells were stained with CellTrace Violet (CTV) proliferation dye and subcultured with or without glutamine (2 mM). Representative plots show CTV staining in EHT-derived $CD43^+$ cells and the gates show the number of divisions, with the percentage of cells in each gate indicated right below. See bar graphs in **Fig. 2a**.

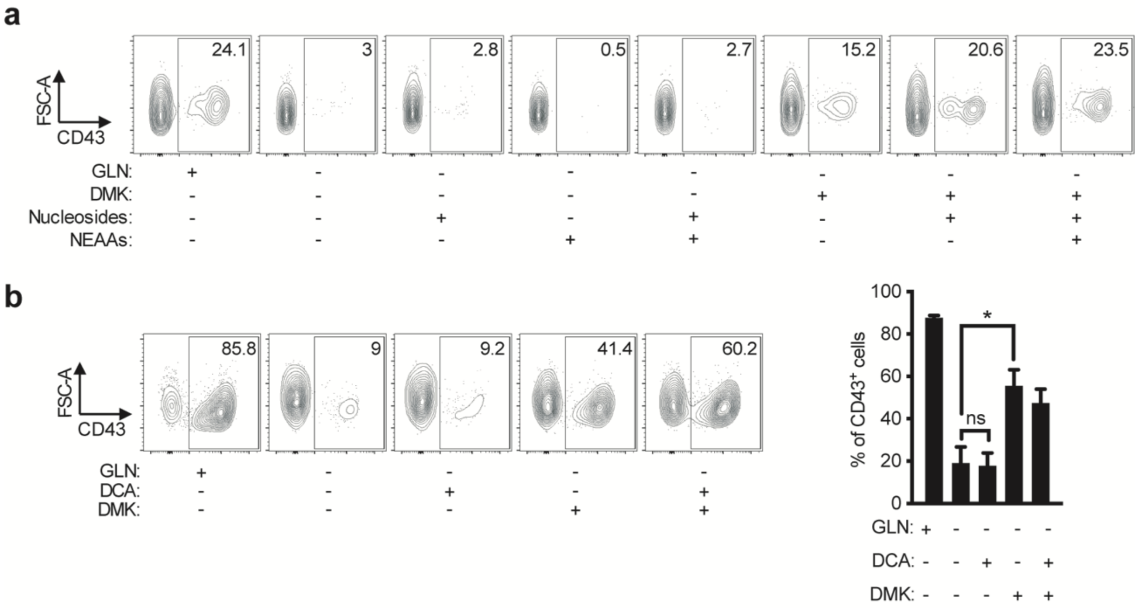

Figure S2. Hematopoietic differentiation of HE requires DMK in the absence of glutamine

(**a**) FACS-sorted HE cells were subcultured in glutamine-free medium with the indicated compounds: DMK (1.75 mM), Nucleosides or NEAAs. Subculture day 3 representative plots for FSC-A/CD43 are shown. (**b**) FACS-sorted HE cells were subcultured in glutamine-free medium with the indicated compounds: DMK (1.75 mM) or DCA (3 mM). Representative FSC-A/CD43 plots and bar graphs of percentages of cells expressing CD43 ± s.e.m. at day 6 of subculture are depicted (n=3, paired *t*-tests). ns, not significant, **p<0.01
